# Uterine Ichthyosis Associated With a High-Grade Squamous Lesion: A Case Report

**DOI:** 10.7759/cureus.71123

**Published:** 2024-10-09

**Authors:** Daniele Camila Maltauro, Tiago Selbach Garcia, Eduardo O Paese, Maria Eduarda Binda, Lucas Coelho, Márcia Luiza Appel

**Affiliations:** 1 Obstetrics and Gynecology, Hospital de Clínicas de Porto Alegre, Porto Alegre, BRA; 2 Gynecologic Oncology, Hospital de Clínicas de Porto Alegre, Porto Alegre, BRA; 3 Medicine, Pontifícia Universidade Católica do Rio Grande do Sul, Porto Alegre, BRA; 4 Medicine, Universidade de Caxias do Sul, Caxias do Sul, BRA

**Keywords:** abnormal uterine bleeding, endometrial lesions, high-grade dysplasia, keratinized epithelium, premalignant endometrial lesions, squamous metaplasia, uterine ichthyosis

## Abstract

Uterine ichthyosis is a rare condition characterized by the extensive replacement of the endometrial surface with keratinized squamous epithelium. While generally benign, it can be associated with premalignant or malignant lesions, making its management critical. Here, we present a case of a 66-year-old patient diagnosed with uterine ichthyosis, highlighting the presence of high-grade dysplasia within the metaplastic squamous epithelium. This finding emphasizes the necessity for careful evaluation and appropriate surgical treatment to rule out potential associated neoplasms. Given the rarity of uterine ichthyosis and its implications for patient management, further research is warranted to establish clear guidelines for diagnosis and treatment. This case underscores the importance of ongoing clinical vigilance and interdisciplinary collaboration in managing patients with this condition.

## Introduction

Ichthyoses are a heterogeneous group of keratinization disorders characterized by visible skin peeling and thickening of the keratinized layer (hyperkeratosis) [1]. Uterine ichthyosis, a rare condition where the endometrial lining is replaced by stratified squamous epithelium, represents a distinct subtype within this category [2]. The etiology of uterine ichthyosis remains poorly defined, with current theories suggesting a possible link to chronic inflammation and infection [3]. Despite being generally benign, uterine ichthyosis can occasionally be associated with malignant neoplasms, highlighting the need for careful clinical monitoring [4].

## Case presentation

A 66-year-old patient, who had undergone menopause at the age of 45 years and had no significant comorbidities, presented with pelvic pain and foul-smelling vaginal discharge for the past four years. On examination, a foul-smelling purulent vaginal discharge was identified, emerging from the external cervical os, along with an extensive area of periorificial leukoplakia on the cervix. Cervical and endometrial biopsies were performed, revealing abundant cornified material with parakeratosis, purulent neutrophilic exudate, and chronic endometritis. Bacteriological examination identified *Morganella morganii* and *Streptococcus anginosus*.

**Figure 1:**
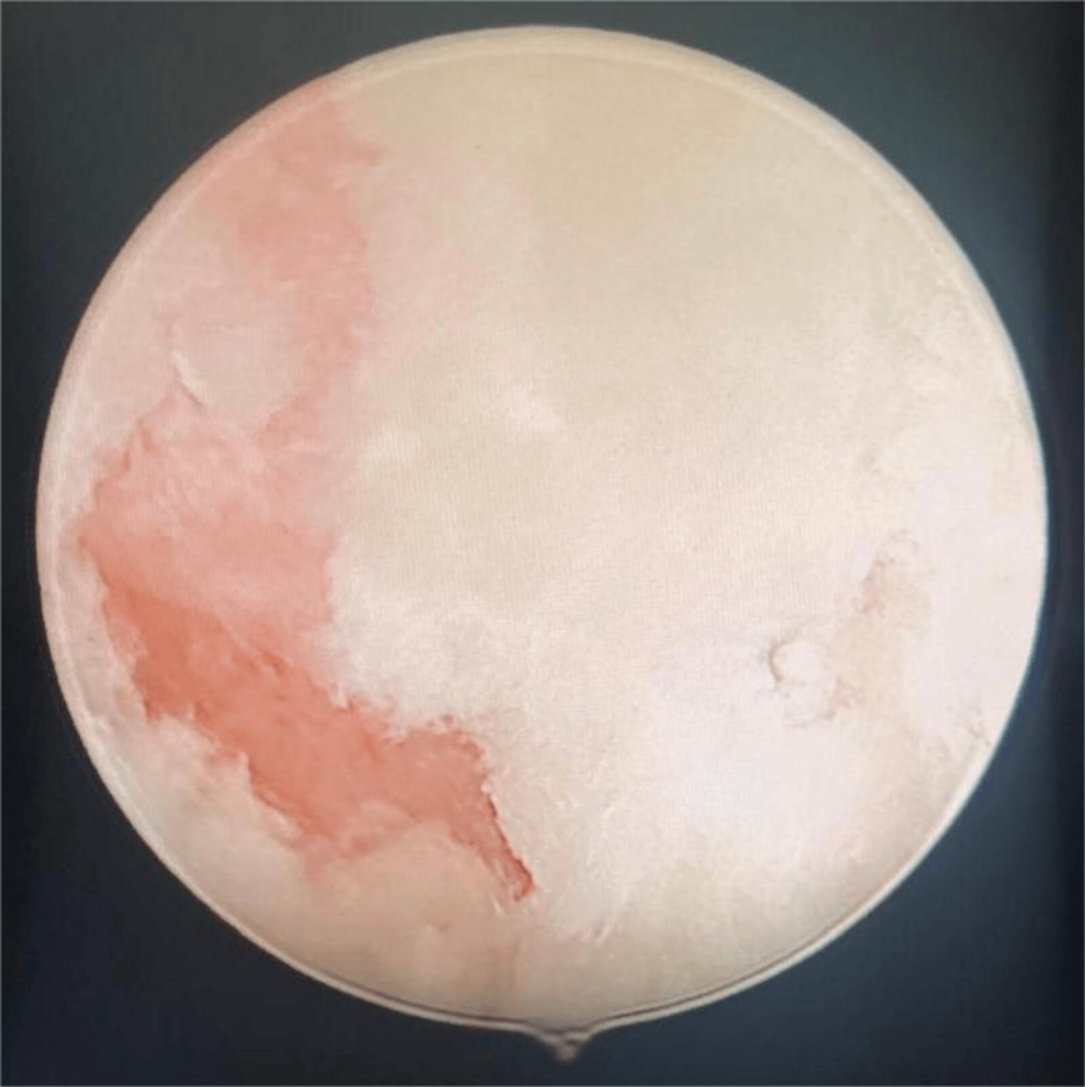
View of the uterine cavity following curettage, revealing a significant amount of whitish, keratinized material.

After 14 days of antibiotics, with partial clinical improvement, the patient underwent cone biopsy, hysteroscopy, and endometrial curettage. Imaging studies, specifically hysteroscopy, and clinical examination revealed a significant amount of whitish material in the uterine cavity and leukoplakia plaques on part of the cervix (Figure 1). Cervical and endometrial biopsies were performed. The anatomopathological findings indicated squamous metaplasia of the endometrium, characterized by extensive keratinization and a predominance of mature epithelium. Focal high-grade dysplasia was observed within the squamous epithelium, but there was no evidence of stromal invasion. The histological appearance suggested uterine ichthyosis with dysplasia, necessitating further sampling to exclude endometrial or cervical neoplasia.

**Figure 2:**
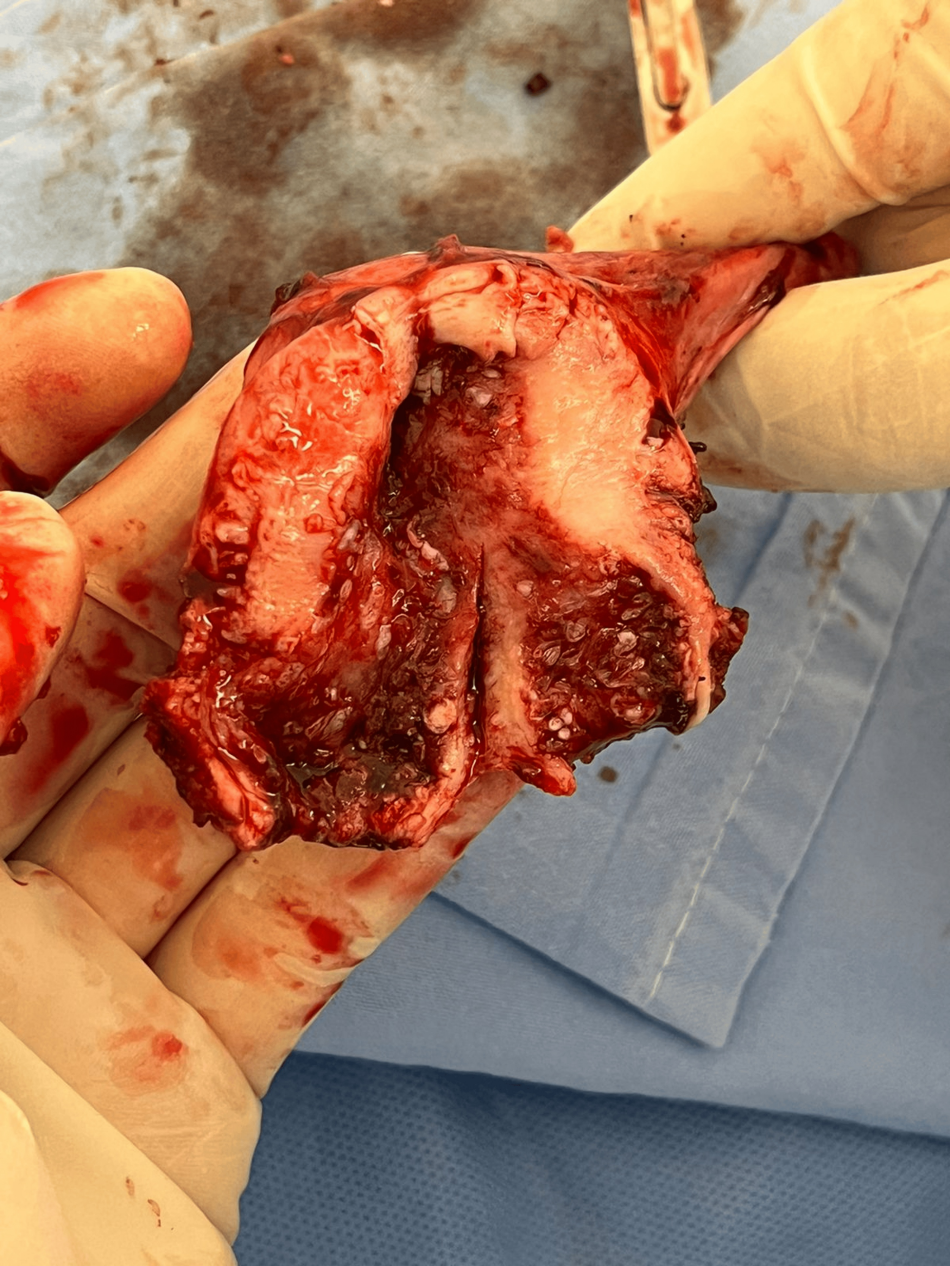
Macroscopic view of the opened uterus showing white-grayish scale-like formations.

Due to the potential association of uterine ichthyosis with endometrial and cervical neoplasms and the persistence of symptoms, the patient underwent laparoscopic hysterectomy with bilateral salpingo-oophorectomy. Macroscopically, the uterus was small and appeared normal, as did the fallopian tubes and ovaries. Upon opening the specimen, white-grayish scale-like formations were observed in the endometrium and endocervix (Figure 2). The histopathological examination confirmed the diagnosis of uterine ichthyosis and showed no signs of malignancy. Specifically, it reported chronic cervicitis characterized by multifocal erosions and extensive keratinization of the squamous mucosa, with thick hyperkeratosis and a granular layer. Importantly, no dysplasia was identified in the squamous epithelium. Additionally, the findings noted extensive squamous metaplasia occupying both the cervical canal and endometrial cavity. The postoperative course was uneventful, and after six months, the patient demonstrated good postoperative recovery and complete resolution of the reported vaginal discharge.

## Discussion

The replacement of extensive areas of the endometrium with stratified squamous epithelium is a rare condition known as uterine ichthyosis [2,3,5,6]. The term "uterine ichthyosis" was first described in 1885 by Zeller to refer to extensive squamous metaplasia of the endometrial surface following the iatrogenic introduction of caustic substances, such as formalin or iodine [5,7]. The etiology of this rare condition is not well understood and appears to be related to chronic infection and inflammation [3,6,7]. Some authors suggest that cervical stenosis, secondary to menopausal cervical atrophy or even a sequel of prior surgical manipulation, may lead to persistent endometrial inflammatory changes, including infection and pyometra, and squamous metaplasia of the endometrium [6]. Notably, in our patient, the presence of chronic cervicitis and endometritis suggests that inflammation may be an etiological factor.

Regarding the clinical presentation, as observed in the patient described above, the most common symptoms reported in the literature include abnormal uterine bleeding, abdominal and pelvic pain, and purulent vaginal discharge in postmenopausal women [7,8].

Although uterine ichthyosis is considered a benign condition, literature reports simultaneous occurrences with dysplastic or malignant changes [3,7,9]. Primary squamous cell carcinoma of the endometrium, a rare neoplasm, has been found to be associated with uterine ichthyosis in some literature [6,9,10]. In 2008, Murhekar et al. suggested the possibility of malignant degeneration of uterine ichthyosis into squamous cell carcinoma [9]. Bagga et al. proposed a sequence of changes in the endometrial lining with squamous metaplasia, followed by dysplasia and overtly invasive carcinoma [10]. Cases of underlying or associated endometrial adenocarcinoma with uterine ichthyosis have also been described [3,5], as well as cases of cervical neoplasia [4]. However, in the context of differential diagnosis of primary squamous tumors of the endometrium associated with ichthyosis, it is important to consider the following pathological criteria defined by Fluhmann in 1953: (i) no evidence of coexisting endometrial adenocarcinoma or squamous cell carcinoma of the cervix, (ii) no connection between the endometrial tumor and the squamous epithelium of the cervix, and (iii) no relationship or connection between any cervical carcinoma in situ and the endometrial tumor [11]. Otherwise, it must be considered that dysplastic or neoplastic events in the endometrial epithelium may be a result of the extension or metastasis of cervical disease (vertical or horizontal carcinogenesis theory of endometrial squamous tumors) [12]. In cases of extension from the cervix, there would also be signs of HPV association, such as koilocytic changes and positive virus testing, which were not described in cases of endometrial squamous cell carcinoma associated with uterine ichthyosis, and positivity for p16 in the affected ichthyosis tissue is not expected [4,9]. In the present case, dysplastic or neoplastic changes of the cervix were excluded. Therefore, the presence of high-grade dysplasia in the metaplastic squamous endometrial epithelium supports the association between ichthyosis and dysplasia and a possible progression to neoplasia.

In suspected cases of uterine ichthyosis, a detailed evaluation of the cervix and obtaining endometrial samples to rule out the presence of coexisting neoplasms is recommended [4]. It is important to note that even procedures such as curettage may have low sensitivity in detecting underlying neoplasms associated with ichthyosis [2,5,6]. Given the uncertain malignant potential of uterine ichthyosis and its association with other lesions, treatment options may vary. While hysterectomy is considered for patients with completed childbearing, it should be approached with caution, especially in cases where the condition appears to be a secondary change due to inflammation. More research and established guidelines are needed to support definitive treatment recommendations [2,3,6,8].

## Conclusions

Currently, due to the rarity of the condition, there is no consensus on the ideal management of uterine ichthyosis. Although the condition is not classified as a pre-malignant lesion with a clear potential for malignant transformation, it is frequently associated with primary squamous cell carcinoma of the endometrium and other gynecological neoplasms. In this case, the inflammation was likely secondary to chronic cervicitis. This underscores the importance of careful evaluation and appropriate surgical treatment to rule out possible associated neoplasms and highlights the need for further studies to define management and follow-up strategies for patients with uterine ichthyosis.
